# Supplementary material for: Causal Relationship between Aging and Anorexia Nervosa: A White-Matter-Microstructure-Mediated Mendelian Randomization Analysis
Source: Biomedicines. 2024 Aug 16;12(8):1874. doi: 10.3390/biomedicines12081874 (PMC11351342; doi:10.3390/biomedicines12081874)
Supplement: Supplementary file 1 [file biomedicines-12-01874-s001.zip › Supplementary Figure/Figure S4/Singe SNP plot of MR analysis for TL to ubm-b-1833.pdf]

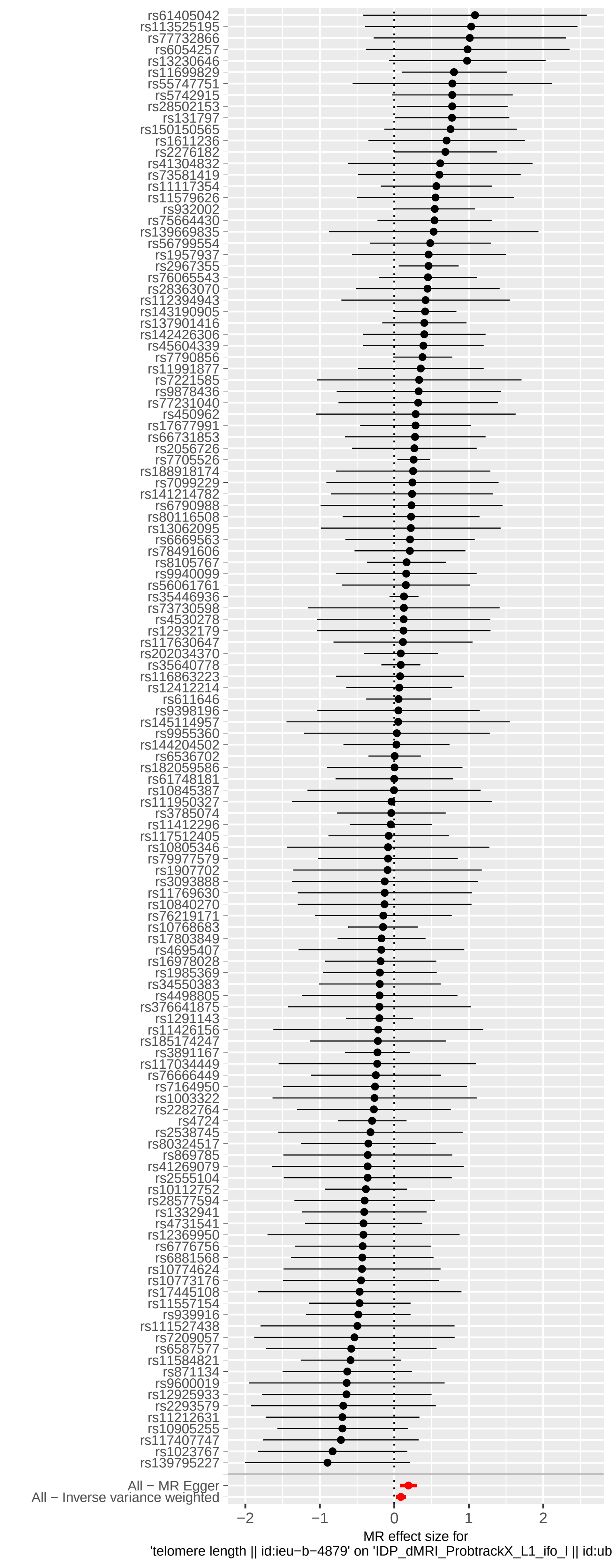
rs61405042
rs113525195
rs77732866
rs6054257
rs13230646
rs11699829
rs55747751
rs5742915
rs28502153
rs131797
rs150150565
rs1611236
rs2276182
rs41304832
rs73581419
rs11117354
rs11579626
rs932002
rs75664430
rs139669835
rs56799554
rs1957937
rs2967355
rs76065543
rs28363070
rs112394943
rs143190905
rs137901416
rs142426306
rs45604339
rs7790856
rs11991877
rs7221585
rs9878436
rs77231040
rs450962
rs17677991
rs66731853
rs2056726
rs7705526
rs188918174
rs7099229
rs141214782
rs6790988
rs80116508
rs13062095
rs6669563
rs78491606
rs8105767
rs9940099
rs56061761
rs35446936
rs73730598
rs4530278
rs12932179
rs117630647
rs202034370
rs35640778
rs116863223
rs12412214
rs611646
rs9398196
rs145114957
rs9955360
rs144204502
rs6536702
rs182059586
rs61748181
rs10845387
rs111950327
rs3785074
rs11412296
rs117512405
rs10805346
rs79977579
rs1907702
rs3093888
rs11769630
rs10840270
rs76219171
rs10768683
rs17803849
rs4695407
rs16978028
rs1985369
rs34550383
rs4498805
rs376641875
rs1291143
rs11426156
rs185174247
rs3891167
rs117034449
rs76666449
rs7164950
rs1003322
rs2282764
rs4724
rs2538745
rs80324517
rs869785
rs41269079
rs2555104
rs10112752
rs28577594
rs1332941
rs4731541
rs12369950
rs6776756
rs6881568
rs10774624
rs10773176
rs17445108
rs11557154
rs939916
rs111527438
rs7209057
rs6587577
rs11584821
rs871134
rs9600019
rs12925933
rs2293579
rs11212631
rs10905255
rs117407747
rs1023767
rs139795227
All − MR Egger
All − Inverse variance weighted
−2
−1
0
1
2
MR effect size for
'telomere length || id:ieu−b−4879' on 'IDP_dMRI_ProbtrackX_L1_ifo_l || id:ub
